# Accessory gene regulator (*agr*) dysfunction was unusual in *Staphylococcus aureus* isolated from Chinese children

**DOI:** 10.1186/s12866-019-1465-z

**Published:** 2019-05-14

**Authors:** Xin Yang, Fang Dong, Suyun Qian, Lijuan Wang, Yingchao Liu, Kaihu Yao, Wenqi Song, Jinghui Zhen, Wei Zhou, Hong Xu, Hongyan Zheng

**Affiliations:** 1 Pediatric Intensive Care Unit, Beijing Children’s Hospital, Capital Medical University, National Center for Children’s Health, No.56 Nan-Li-Shi Road, Beijing, 100045 China; 2 Bacteriology Laboratory, Beijing Children’s Hospital, Capital Medical University, National Center for Children’s Health, Beijing, 100045 China; 3 MOE Key Laboratory of Major Diseases in Children, National Key Discipline of Pediatrics (Capital Medical University), National Clinical Research Center for Respiratory Diseases, Beijing Key Laboratory of Pediatric Respiratory Infection Diseases, Beijing Pediatric Research Institute, Beijing Children’s Hospital, Capital Medical University, National Center for Children’s Health, Beijing, 100045 China; 4 Present address: No. 56 Nan-li-shi Road, Beijing, 100045 China

**Keywords:** *Staphylococcus aureus*, Accessory gene regulator, Children, China

## Abstract

**Background:**

*Staphylococcus aureus* (*S. aureus*) with accessory gene regulator (*agr*) dysfunction occurs in health care settings. This study evaluated the prevalence and the molecular and drug resistance characteristics of *S. aureus* with dysfunctional *agr* in a pediatric population in Beijing, China.

**Results:**

A total of 269 nonduplicate *S. aureus* clinical isolates were isolated from Beijing Children’s Hospital, including 211 methicillin-resistant *S. aureus* (MRSA) from September 2010–2017 and 58 methicillin-sensitive *S. aureus* (MSSA) from February 2016–2017. Only 8 MRSA and 2 MSSA isolates were identified as *agr* dysfunction, and the overall prevalence rate was 3.7%. For MRSA isolates, ST59-SCC*mec* IV and ST239-SCC*mec* III were the most common clones, and the prevalence rate of *agr* dysfunction in ST239-SCC*mec* III isolates (17.39%) was significantly higher than in ST59-SCC*mec* IV (1.69%) and other genotype strains (*P* = 0.006). Among the *agr* dysfunctional isolates, only one MRSA ST59 isolate and one MSSA ST22 isolate harbored *pvl*. No significant difference was detected between *agr* dysfunction and *agr* functional isolates regarding the biofilm formation ability (*P* = 0.4972); however, 9/10 *agr* dysfunctional isolates could effectuate strong biofilm formation and multidrug resistance. Among MRSA, the non-susceptibility rates to ciprofloxacin, gentamicin, and trimethoprim-sulfamethoxazole were significantly higher in *agr* dysfunctional isolates than in isolates with functional *agr* (*P* < 0.05). Two isolates belonging to ST239 had no mutations in *agr* locus, but a synonymous mutation was found in *agrA* in another ST239 isolate. The inactivating mutations were detected in other seven *agr* dysfunctional isolates. The variants were characterized by non-synonymous changes (*n* = 5) and frameshift mutations (insertions, *n* = 2), which mainly occurred in *agrC* and *agrA*.

**Conclusions:**

The results showed that *agr* dysfunctional *S. aureus* was not common in Chinese children, and ST59-SCC*mec* IV was associated with lower prevalence of *agr* dysfunction as compared to ST239-SCC*mec* III isolates. The *agr* dysfunctional isolates were healthcare-associated, multidrug resistant and form strong biofilm, which suggested that *agr* dysfunction might offer potential advantages for *S. aureus to* survive in a medical environment.

## Background

*Staphylococcus aureus* (*S. aureus*) continues to be a leading cause of both community-and healthcare-associated infections, including skin and soft tissue infections, bacteremia, pneumonia, osteomyelitis, and endocarditis. Virulence factors play a major role in the pathogenesis of *S. aureus*, such as Panton-Valentine leukocidin (PVL). PVL is a pore-forming exotoxin composed of LukS-PV and LukF-PV. The PVL-producing strains have been associated with the onset of skin and soft tissue infections (SSTIs) and can also cause severe invasive infections (necrotizing pneumonia, etc) [1]. In addition, *S. aureus* can form biofilms not only in biological samples and surfaces of medical devices but also in tissues [2]. Biofilm can protect *S. aureus* from the damage of antibiotics and the host immune system [3]. Subsequently, the successful eradication of *S. aureus* infections is difficult, rendering biofilm as a vital factor in chronic infections.

The accessory gene regulator (*agr*)-mediated quorum sensing plays a major role in staphylococcal pathogenesis, which can downregulate the expression of cell surface-associated proteins (microbial surface components recognizing adhesive matrix molecules [MSCRAMMs]) and upregulate the expression of extracellular toxin (hemolysins, enterotoxins, extracellular proteases, etc.) [4]. The regulation of virulence factors by *agr* is important for disease progression. Several studies demonstrated that genetically engineered *agr*-knockout strains had attenuated virulence in animal models of skin and soft tissue infections, pneumonia, infective endocarditis, arthritis, and osteomyelitis [5–10].

However, recent evidence indicated that *agr* dysfunction exists extensively in healthcare settings (13–82%) [11–14], which might be attributed to the *agr* dysfunction that confers a potential advantage for *S. aureus* in the current medical environment [15]. For example, *agr* dysfunction has been linked to attenuated vancomycin activity; both laboratory-derived vancomycin-intermediate *S. aureus* (VISA) and clinical VISA isolates developed during vancomycin therapy often exhibit as *agr* dysfunction [16]. In addition, infections, such as bacteremia and bone and joint infection, caused by strains with dysfunctional *agr* often manifest as a chronic course and result in adverse outcomes [17–19]. Thus, understanding the prevalence of *agr* dysfunction in a specific area is essential.

Herein, we conducted a molecular epidemiology study in Beijing Children’s Hospital in China. The primary objectives of the present study were as follows: (1) to detect the prevalence of *agr* dysfunction in MRSA and MSSA isolates; (2) to explore the molecular characteristics, *pvl* carriage rate, biofilm formation ability, and antibiotic susceptibility of *S. aureus* isolated from pediatric patients; these indicators were compared mainly based on *agr* functionality.

## Methods

### Bacterial isolates

This study was approved by the Ethics Committee of Beijing Children’s Hospital affiliated to the Capital Medical University (No. 2016–93, 23/06/2016), and obtained clearance from the Institutional Biosafety Committee (IBSC) ([2017] No.43). *S. aureus* strains were collected and identified as follows. If the clinical samples (blood, pleural effusion, and joint effusion, etc.) were obtained from steril specimens (blood, pleural effusion, bone marrow, cerebrospinal fluid, joint effusion, seroperitoneum, etc), the bacterial growth in the culture medium could be directly identified by VITEK® MS system (BioMérieux, France). If the clinical samples were obtained from the non-sterile specimens (respiratory tract, skin, etc.), several suspected colonies were selected according to the morphological characteristics and identified by VITEK® MS system. The coagulase test and detection of *nuc* gene were employed to identify *S. aureus* as described previously by Kateete et al. [20] and Petersson et al. [21], respectively; these isolates were further confirmed to be *S. aureus*. The MRSA isolates were screened by cefoxitin disc (30 mg, Oxoid) diffusion test, while the polymerase chain reaction (PCR) was employed for the detection of the *mecA* gene [22]. In the case of strains isolated from the same patient, if the genotyping studies revealed identical genotype, only one of them was included in the study, which ensured that all strains involved in the current study were non-repetitive. All the strains were preserved at − 80 °C in a bacterial cryopreservation reagent comprised of 2.5% TSB (w/v), 16.7% glycerol (v/v), and 66.7% sterile horse serum (v/v).

The clinical data of children, including age, sex, medical history, isolation site, infection sites, and medication use, were collected. *S. aureus* infections were categorized as healthcare-associated (HA) or community-associated (CA) according to the epidemiology definitions established previously [23].

### Delta-hemolysin expression

RNAIII is the major effector molecule of *agr* system, and also encodes the gene for delta-hemolysin (*hld*) [24]. Thus, δ-hemolysin production was used to assess the function of *agr* operon. The expression of δ-hemolysin was determined using *S. aureus* RN4420, which produced only β-hemolysin without the interference of α- or δ- hemolysins. Furthermore, β-hemolysin and δ-hemolysin have synergetic effects, while β-hemolysin inhibits lysis by α-hemolysin [25]. Therefore, the presence of enhanced hemolysis within the β-hemolysin zone of RN4220 indicates the production of δ-hemolysin by the test strains.

### *agr* sequencing

The *agr* locus of non-hemolytic isolates was amplified using the method reported by Robinson et al. [26]. In addition, *agr*-w1f (5′-CCATTTgCCCAATTCATTC-3′) was used for sequencing the PCR product amplified by *agr*X1F (5′-TCGTATAATGACAGTGAGGAGAGT-3′) and *agr*CD2434dn (5′-TAATACCAATACTGCGACTT-3′). The resultant sequences were compared with the known *agr* sequences of *S. aureus* strain from the appropriate *agr* specificity group and sequence types (STs), including NCTC8325 (CC8) (CP000253.1), SA957 (ST59) (NC_022442.1), ST398 (NC_017333.1), N315 (*agr* group II), and H-EMRSA-15 genome (ST22) (CP007659.1).

### Molecular genotyping analysis

Multilocus sequence typing (MLST) was performed as described by Enright et al. [27]. The allelic profiles (allele numbers) and ST types were determined based on the MLST database (http://saureus.mlst.net/). The staphylococcal protein A (*spa*) gene repeat region was amplified and sequenced as described previously [28], and the sequencing data were submitted to the *S. aureus spa* type database (http://spaserver.ridom.de) to determine the *spa* type. *Agr* typing was assigned by multiplex PCR according to the method described by Gilot et al. [29]. COL (*agr* I), N315 (*agr* II), TY114 (*agr* III), and A920210 were used as positive controls.

The staphylococcal cassette chromosome *mec* (SCC*mec*) types of MRSA isolates were determined using a multiplex PCR as described previously [30]. The reference strains used for SCC*mec* typing included NCTC10442 (SCC*mec* I), N315 (SCC*mec* II), 85/2082 (SCC*mec* III), JCSC4744 (SCC*mec* IV), and IMVS 67 (SCC*mec* V).

### Detection of *pvl* gene

The detection of *pvl* was carried out using primers and conditions as described by Jarraud et al. [31]. ATCC25923 was used as a positive control.

### Biofilm formation assays

Tissue culture plate method (TCP) was used to assess the biofilm forming ability of the nonhemolytic strains, as described in our previous study [32]. Briefly, overnight cultures in tryptic soy broth (TSB) (OXOID, USA) containing 0.25% glucose were adjusted to 10^6^ CFU/mL. Then, 0.2 mL cell suspension was inoculated into each well of 96-well flat-bottom plates (Corning Costar #3599, USA) at 37 °C for 48 h. Subsequently, the wells were washed two times with normal saline, fixed by methanol for 15 min, stained with 0.1% crystal violet for 5 min, rinsed, and air-dried. The stained biofilm was solubilized with 33% glacial acetic acid, and the optical density (OD) was measured at 590 nm using on a CLARIOstar Microplate reader (BMG LABTECH, Germany). Each isolate was tested in triplicates. The negative control wells contained only the broth. The cut-off OD value (ODc) was defined as an average OD of negative control with three times of standard deviation. The biofilm formation ability was classified as negative (OD ≤ ODc), weak (ODc < OD ≤ 2ODc, WBF), moderate (2ODc < OD ≤ ODc, MBF), and strong (4ODc < OD, SBF).

### Antimicrobial susceptibility testing

Antimicrobial susceptibility testing to 12 antimicrobial agents (penicillin G, oxacillin, erythromycin, clindamycin, tetracycline, gentamicin, chloramphenicol, ciprofloxacin, rifampin, linezolid, vancomycin, mopiroxacin; National Institutes for Food and Drug Control, China) were performed by agar dilution method as described by Wiegand et al. [33] with slight modification. Mueller-Hinton Agar (MHA; OXOID CM0337B, UK) medium without cation-adjustments were prepared and autoclaved according to the manufacture’s instructions. The antibacterial drugs were diluted for usage according to the Clinical and Laboratory Standards Institute (CLSI) guidelines [34]. Appropriate MHA medium was cooled to about 50 °C and poured to the 15 × 100 mm petri dish to produce the required depth of 3–4 mm. The final antibiotic concentration for each drug ranged from 0.032–256 mg/L and three control agar plates were without any antibiotic. The inoculation was carried out using a multipoint inoculator. Bacterial suspension, at a density of 1 × 10^7^ CFU/mL, was inoculated to the agar plates starting from the lowest concentration. Then, the inoculum spots were dried at room temperature before inverting the plates and incubated at 35 °C for 16–20 h before obtaining the minimal inhibitory concentration (MIC). In addition, the E-test method was used to determine the MIC of all isolates to sulphamethoxazole/trimethoprim (SXT) (BioMeriuex, France). MHA plates were inoculated by streaking the standardized inoculums (0.5 McFarland, about 1.5 × 10^8^ CFU/mL with a sterile swab. The SXT E-test strips (BioMeriuex) were placed on the plates, followed by incubation at 35 °C for 16–20 h.

The MIC reading for both E-test and agar dilution method was conducted independently by a senior experimenter, with the result confirmed by a second reader. The results of MIC were interpreted according to the CLSI breakpoints for *Staphylococcus* spp. [34]. *S. aureus* ATCC29213 was used as quality control. Multidrug resistance (MDR) was defined as isolates resistant to ≥3 classes of non-β-lactam antimicrobials for MRSA, and resistant to ≥3 classes of antibiotics including β-lactam antibiotics for MSSA.

### Statistical analysis

SAS JMP Statistical Discovery v11.0 was used for statistical analysis. Chi-squared (χ^2^) test or Fisher’s exact test was used to analyze the categorical variables, and Wilcoxon rank sum test was used to compare the biofilm formation ability between the two groups. *P* < 0.05 was considered as statistically significant.

## Results

### Clinical characteristics

A total of 269 non-duplicate *S. aureus* clinical isolates were collected from Beijing Children’s Hospital, including 211 MRSA from September 2010–2017 and 58 MSSA from February 2016–2017. These strains were isolated from several clinical sources, including respiratory tract (6 from throat swab, 69 from sputum, and 37 from bronchial alveolar lavage fluid), skin and soft tissue (38 from pus, 20 from secretions of omphalitis, 10 from skin secretions, 17 from wound surface, 6 from eye secretions, and 2 from ear secretions), sterile sites (48 from blood, 6 from pleural effusion, 3 from bone marrow, 3 from cerebrospinal fluid, 2 from joint effusion, and 1 from seroperitoneum), and midstream urine (1 isolate). The characteristics of patients (107 females and 162 males; median age: 11.9 months) from whom samples were collected are shown in Table 1. Approximately 72.86% (196/269) of the patients were < 3-years-old. The proportion of children with community-associated (CA) infections and hospital-associated infections was roughly equivalent (50.19% vs. 49.81%). Then, 9/10 *agr* dysfunctional isolates caused healthcare-associated (HA) infections. A total of 29.74% (80/269) children presented invasive infections. All children included in this study were treated with antibiotics. Vancomycin and linezolid were used in 24.54% (66/269) and 16.73% (45/269) patients, respectively. The median hospital stay was 15 (interquartile range, IQR: 10–24) Days.

**Table 1:** Characteristics of patients for samples

Patient characteristics	Total	Dysfunctional *agr* (*N* = 10)	Functional *agr* (*N* = 159)	*P*-value
Male sex, N (%)	162 (60.22)	5 (50.00)	157 (60.62)	0.5253
Age (months), median (IQR^*a*^)	11.9 (1.27–55.14)	33.27 (13.15–108.38)	11.33 (1.17–54.90)	0.0797
Age distribution				0.1581
≤ 28 days	59 (21.93)	0	59 (22.78)	
29 days–3 years	137 (50.93)	7 (70.00)	130 (50.19)	
4–6 years	28 (10.41)	0	28 (10.81)	
7–15 years	45 (16.73)	3 (30.00)	42 (16.22)	
Origin, N (%)				0.0102
CA	135 (50.19)	1 (10.00)	134 (51.74)	
HA	134 (49.81)	9 (90.00)	125 (48.26)	
Disease				0.2900
Invasive infection^*b*^, N (%)	80 (29.74)	1 (10.00)	9 (90.00)	
Non-invasive infection^*c*^, N(%)	189 (70.26)	79 (30.50)	180 (69.50)	
Vancomycin treatment, N (%)	66 (24.54)	2 (20.00)	64 (24.71)	1.0000
Linezolid treatment, N (%)	45 (16.73)	2 (20.00)	43 (16.60)	0.6758
Hospitalization-Median (IQR)	15 (10–24)	21 (10.25–30.25)	14 (10–23)	0.4000

^*a*^*IQR* interquartile range

^*b*^*SSTI* Including skin and soft tissue infection, *BSI* bloodstream infection, *CNSI* central nervous system infection, *IE* infective endocarditis, *BJI* bone and joint infection, *SP* severe pneumonia, *AI* intra-abdominal infection. Details were as follows: SSTI (1 case), AI (1 case), CNSI (1 case), BJI (3 cases), SP (11 cases), BSI (11 cases), BSI + SSTI (16 cases), BSI + AI (1 case), BSI + IE (4), BSI + BJI (6 cases), BSI + CNSI (2 cases), BSI + SP (5 cases), BJI + SSTI (1 case), SP + SSTI (1 case), SP + BJI (1 case), BSI + SSTI+CNSI (1 case), BSI + CNSI+IE (1 case), BSI + SP + CNSI (1 case), BSI + SP + SSTI (3 cases), BSI + BJI + SSTI (7 cases), BSI + BJI + SSTI+CNSI (1 cases), BSI + SP + SSTI+CNSI (1 case)

^c^Including SSTI (83 cases), pneumonia (103 cases), pneumonia+SSTI (2 cases), urinary system infection (1 case)

### Molecular typing and virulence characteristics

**Fig. 1:**
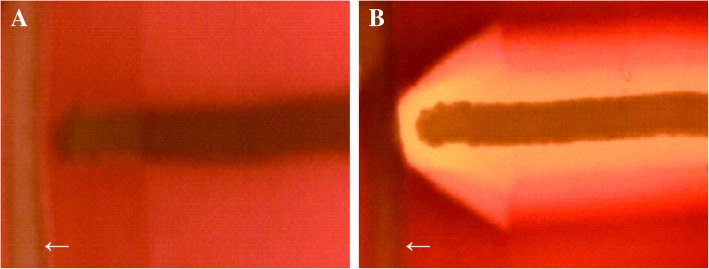
δ-hemolysin assays. **a** δ-hemolysin-negative; **b** δ-hemolysin-positive (LAC USA 300). The arrows showed RN4220

Among the 269 isolates, only 3.79% (8/211) MRSA and 3.45% (2/58) MSSA isolates were identified with *agr* dysfunction (no apparent hemolytic activity as shown in Fig. 1), and the overall prevalence rate was 3.71% (10/269).

The molecular characteristics of MRSA and MSSA stratified by *agr* functionality were shown in Table 2 and Table 3, respectively. ST59-SCC*mec* IV-t437/t441-*agr* I (49.29%, 104/211) and ST239-SCC*mec* III-t030/t037-*agr* I (9.00%, 19/211) were the most common MRSA clones. The prevalence rate of *agr* dysfunction in ST239-SCC*mec* III isolates (17.39%, 4/23) was significantly higher than in ST59-SCC*mec* IV (1.69%, 2/118) and other genotype strains (2.86%, 2/70) (*P* = 0.006) (Table 1). For MSSA isolates, the top 4 genotypes were ST22-t309-*agr* I (15.52%, 9/58), ST398-t571-*agr* I (10.34%, 6/58), ST5-t002-*agr* II (6.90%, 4/58), ST6-t701-*agr* I (6.90%, 4/58), and ST188-t189-*agr* I (6.90%, 4/58).

**Table 2:** Genotypic characteristics of MRSA isolates stratified by *agr* functionality

Genotype	No. of solates (%)	*agr*		*pvl* (+), N (%)
		Dysfunction	Function	
Total	211	8 (3.79)	203 (96.21)	75 (35.55)
Origins				
Community-associated	104 (49.29)	0	104 (100.00)	44 (42.30)
Hospital-associated	107 (50.71)	8 (7.47)	99 (92.52)	31 (28.97)
MLST				
1	8 (3.79)	0	8 (100.00)	2 (25.00)
22	7 (3.32)	0	7 (100.00)	7 (100.00)
59	128 (60.66)	2 (1.56)	126 (98.44)	49 (38.28)
88	8 (3.79)	0	8 (100.00)	5 (62.50)
239	27 (12.80)	4 (14.81)	23 (85.19)	2 (7.41)
Others^*a*^	33 (15.64)	2 (6.06)	31 (93.94)	10 (30.30)
SCC*mec*				
I	1 (0.47)	0	1 (100.00)	0
III	26 (12.32)	4 (15.38)	22 (84.62)	2 (7.69)
IV	145 (68.72)	3 (2.07)	142 (97.93)	51 (35.17)
V	32 (15.17)	1 (3.13)	31 (96.87)	19 (59.38)
NT^*b*^	7 (3.32)	0	7 (100.00)	3 (42.86)
*spa* type				
t030	16 (7.58)	0	16 (100.00)	0
t037	7 (3.32)	3 (42.86)	4 (57.14)	1 (14.29)
t309	7 (3.32)	0	7 (100.00)	7 (100.00)
t437	107 (50.71)	2 (1.87)	105 (98.13)	47 (43.92)
t441	14 (6.64)	0	14 (100.00)	7 (50.00)
Others^*c*^	60 (28.43)	3 (5.00)	57 ()95.00)	13 (21.67)
*agr* type				
I	185 (87.68)	7 (3.78)	178 (96.22)	67 (36.22)
II	6 (2.84)	1 (16.67)	5 (83.33)	1 (16.67)
III	17 (8.06)	0	17 (100.00)	7 (41.18)
IV	3 (1.42)	0	3 (100.00)	0
MLST-SCC*mec* type				
ST1-SCC*mec* IV	6 (2.84)	0	6 (100.00)	0
ST22-SCC*mec* V	7 (3.32)	0	7 (100.00)	7 (100.00)
ST59-SCC*mec* IV	118 (55.92)	2 (1.69)	116 (98.31)	44 (37.29)
ST239-SCC*mec* III	23 (10.90)	4 (17.39)	19 (82.61)	1 (4.35)
Others	57 (27.01)	2 (3.51)	50 (96.49)	23 (40.35)
MLST-SCC*mec*-*spa*-*agr* type				
ST59-SCC*mec* IV-t437-*agr* I	92 (43.60)	1 (1.09)	91 (98.91)	36 (39.13)
ST59-SCC*mec* IV-t441-*agr* I	12 (5.69)	0	12 (100.00)	6 (50.00)
ST239-SCC*mec* III-t030-*agr* I	12 (5.69)	0	12 (100.00)	0
ST239-SCC*mec* III-t037-*agr* I	7 (3.32)	3 (42.86)	4 (57.14)	0
ST22-SCC*mec* V-t039-*agr* I	7 (3.32)	0	7 (100.00)	7 (100.00)
Others	81 (38.39)	4 (4.94)	77 (95.06)	26 (32.10)

^*a*^The other MLSTs were ST5 (2 isolates), ST6 (1 isolate), ST9 (1 isolate), ST30 (1 isolate), ST72 (2 isolates), ST97 (1 isolate), ST120 (1 isolate), ST121 (1 isolate), ST375 (1 isolate), ST509 (1 isolate), ST585 (1 isolate), ST630 (1 isolate), ST896 (1 isolate), ST950 (1 isolate), ST965 (2 isolates), ST1224 (1 isolate), ST1295 (1 isolate), ST1296 (1 isolate), ST1777 (1 isolate), ST1821 (1 isolate)

^*b*^Not determined

^*c*^The other spa types were t008 (1 isolate), t011 (1 isolate), t021 (1 isolate), t034 (2 isolates), t062 (2 isolates), t078 (2 isolates), t114 (6 isolates), t127 (3 isolates), t138 (1 isolate), t163 (1 isolate), t172 (4 isolate), t186 (1 isolate), t267 (1 isolate), t318 (1 isolate), t459 (2 isolate), t664 (1 isolate), t895 (2 isolates), t1894 (1 isolate), t1977 (1 isolate), t2270 (1 isolate), t2310 (1 isolate), t2755 (2 isolate), t3401 (1 isolate), t3515 (2 isolates), t3523 (1 isolate), t3590 (1 isolate), t4431 (1 isolate), t4549 (2 isolates), t7617 (1 isolate), t7637 (1 isolate), t8660 (2 isolates), t8723 (1 isolate), t10555 (1 isolate), t12946 (1 isolate), t16365 (1 isolate)

**Table 3:** Genotypic characteristics of MSSA isolates stratified by *agr* functionality

Genotype	No. of solates (%)	No. (%) of isolates		*pvl* positive [No. (%)]
		Dysfunctional *agr*	Functional *agr*	
Total	58	2 (3.45)	56 (96.55)	17 (29.31)
Community-associated	31 (53.45)	1 (3.23)	30 (96.77)	12 (38.71)
Hospital-associated	27 (46.55)	1 (3.70)	26 (96.30)	5 (18.52)
MLST				
5	5 (8.62)	0	5 (100.00)	0
7	5 (8.62)	0	5 (100.00)	1 (20.00)
22	12 (20.69)	1 (8.33)	11 (91.67)	12 (100.00)
25	6 (10.34)	0	6 (100.00)	1 (16.67)
398	10 (17.24)	1 (10.00)	9 (90.00)	1 (10.00)
Others^*a*^	20 (34.48)	0	20 (100.00)	2 (10.00)
*spa* type				
t002	4 (6.89)	0	4 (100.00)	0
t189	4 (6.89)	0	4 (100.00)	0
t309	11 (18.97)	0	11 (100.00)	11 (100.0)
t571	6 (10.34)	1 (16.67)	5 (83.33)	0
t701	4 (6.90)	0	4 (100.00)	0
Others^*b*^	29 (50.00)	1 (3.45)	28 (96.55)	6 (20.69)
*agr* type				
I	46 (79.31)	2 (4.35)	44 (95.65)	16 (34.78)
II	9 (15.52)	0	9 (100.00)	0
III	1 (1.72)	0	1 (100.00)	0
IV	1 (1.72)	0	1 (100.00)	0
NT^*c*^	1 (1.72)	0	1 (100.00)	1 (100.00)
MLST-*spa*-*agr* type				
ST5-t002-*agr* II	4 (6.90)	0	4 (100.00)	0
ST6-t701-*agr* I	4 (6.90)	0	4 (100.00)	0
ST22-t309-*agr* I	9 (15.52)	0	9 (100.00)	9 (100.00)
ST188-t189-*agr* I	4 (6.90)	0	4 (100.00)	0
ST398-t571-*agr* I	6 (10.34)	1 (16.67)	5 (83.33)	0
Others	31 (53.45)	1 (3.23)	30 (96.77)	8 (25.81)

^*a*^The other MLSTs were ST1 (1 isolate), ST6 (4 isolate), ST8 (1 isolate), ST15 (3 isolates), ST25 (6 isolates), ST59 (4 isolates), ST121 (1 isolate), ST188 (4 isolates), ST950 (1 isolate), ST1281 (1 isolate)

^*b*^The other spa types were t034 (3 isolates), t078 (2 isolates), t081 (1 isolate), t084 (3 isolates), t091 (2 isolates), t127 (1 isolate), t163 (1 isolate), t164 (1 isolate), t167 (1 isolate), t310 (1 isolate), t437 (2 isolates), t660 (1 isolate), t796 (2 isolates), t1062 (1 isolate), t1818 (1 isolate), t2092 (1 isolate), t4377 (1 isolate)

^*c*^Not determined

The detection rate of *pvl* was similar in MRSA (35.55%, 75/211) and MSSA (29.31%, 17/58) isolates (*P* = 0.4359). Among MRSA, the *pvl* prevalence rate of ST239-SCC*mec* III isolates (4.35%, 1/23) was significantly lower than that of ST59-SCC*mec* IV (37.3%, 44/118) and other strains (42.86%, 30/70) (*P* = 0.0031). All the ST22 isolates were *pvl*-positive. Among the ten agr dysfunctional isolates, only one MRSA belonging to the ST59-SCC*mec* IV-t437-*agr* I and one MSSA belonging to ST22-t310-*agr* I harbored *pvl*.

### Biofilm formation

**Fig. 2:**
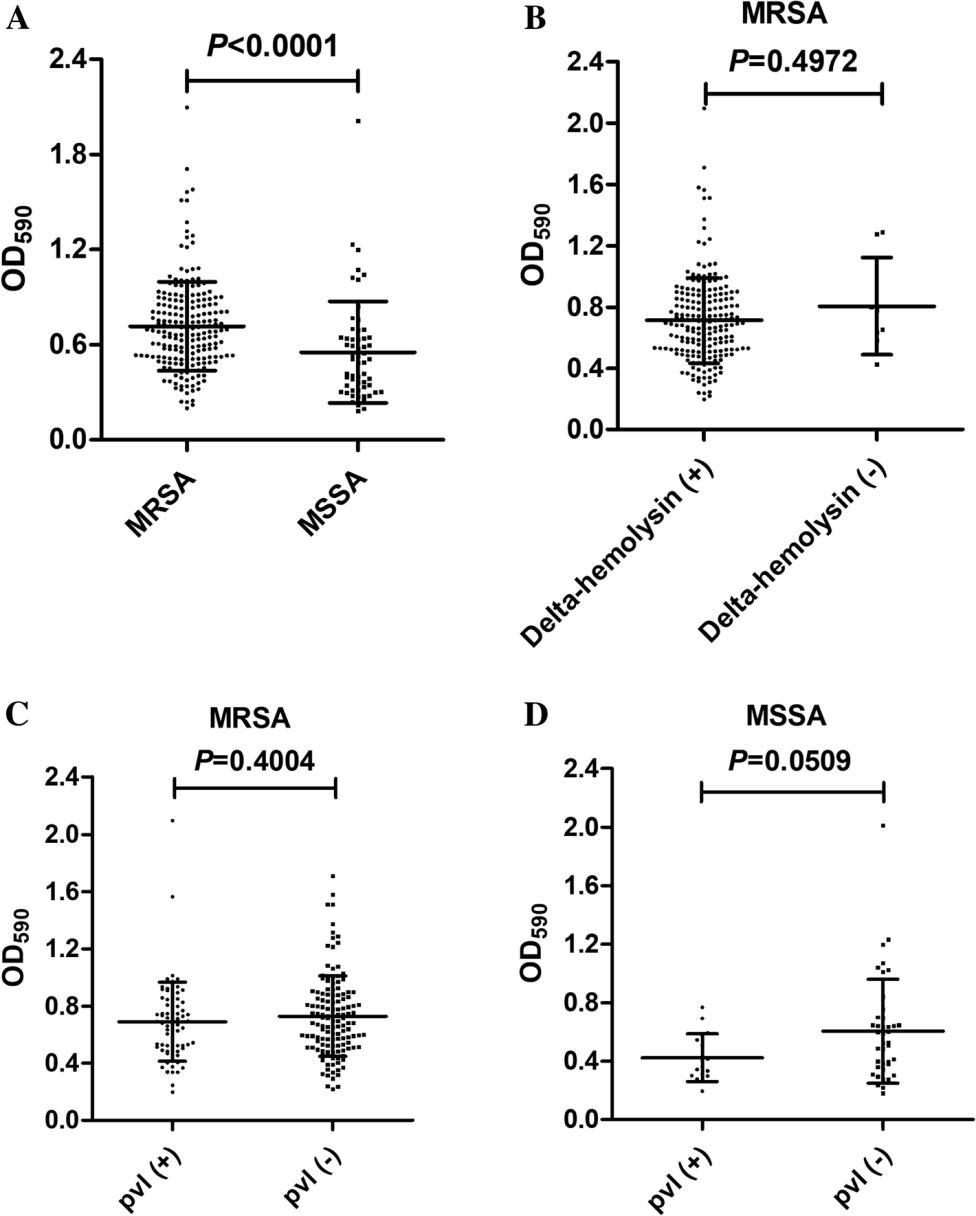
Biofilm formation ability of *S. aureus*. **a** Comparison between MRSA and MSSA isolates. **b** Comparison between *agr* dysfunctional and *agr* functional isolates among MRSA. **c** Comparison between *pvl* (+) and *pvl* (−) isolates among MRSA. **d** Comparison between *pvl* (+) and *pvl* (−) isolates among MSSA. OD_590_, optical density at 590 nm

Table 4 shows that 88.63% (187/211) of MRSA and 56.90% (33/58) of MSSA isolates were strong biofilm formers. However, the biofilm formation ability of MRSA was significantly higher than that of MSSA isolates (*P* < 0.0001) (Fig. 2a). Interestingly, no significant difference was detected between *agr* dysfunction and *agr* functional isolates regarding the biofilm formation ability (*P* = 0.4972) (Fig. 2b); nevertheless, all the eight *agr* dysfunctional MRSA isolates and one MSSA isolate showed strong biofilm formation. Any association between *pvl* and biofilm formation in both MRSA and MSSA isolates was not observed (*P* = 0.4004 and *P* = 0.0509, respectively) (Figs. 2c and d).

**Table 4:** Biofilm formation ability of MRSA and MSSA regarding *agr* functionality [N (%)]

	No. of isolates	WBF	MBF	SBF
MRSA	211	1 (0.47)	23 (10.90)	187 (88.63)
Functional *agr*	203	1 (0.49)	23 (11.33)	179 (88.18)
Dysfunctional *agr*	8	0	0	8 (100.00)
MSSA	58	2 (3.45)	23 (39.66)	33 (56.90)
Functional *agr*	56	2 (3.57)	22 (39.29)	32 (57.14)
Dysfunctional *agr*	2	0	1 (50.00)	1 (50.00)
Total	269	3 (1.11)	48 (17.84)	218 (81.04)

*WBF* weak biofilm formation, *MBF* moderate biofilm formation, *SBF* strong biofilm formation

### Antimicrobial resistance

The results of antimicrobial susceptibility test are shown in Table 5. In this study, the *S. aureus* isolates were sensitive to vancomycin and linezolid, but highly resistant to penicillin (97.03%, 261/269) and erythromycin (98.89%, 266/269). Approximately, 80.09% (169/211) of MRSA and 74.14% (43/58) of MSSA isolates were MDR strains. The non-susceptibility rate of MRSA to oxacillin, clindamycin, tetracycline, and rifampin was significantly higher than that of MSSA isolates (*P* < 0.05). In addition, the non-susceptibility rates to ciprofloxacin, gentamicin, and SXT among MRSA isolates were significantly higher in *agr* dysfunctional isolates than in those with functional *agr*.

**Table 5:** Non-susceptibility rates of *S. aureus* in pediatric population in China [N (%)]

	No. of isolates	PEN	OXA	ERY	CLI	TET	GEN	CHL	RIF	CIP	SXT	MDR
MRSA	211	207 (98.10)	176 (83.41)	208 (98.58)	181 (85.78)	103 (48.82)	38 (18.01)	121 (57.35)	22 (10.43)	65 (30.81)	5 (2.37)	169 (80.09)
Dysfunctional *agr*	8	8 (100.0)	7 (87.50)	8 (100.0)	7 (87.50)	6 (75.00)	6 (75.00)	3 (37.50)	1 (12.50)	7 (87.50)	3 (37.50)	7 (87.50)
Functional *agr*	203	199 (98.03)	169 (83.25)	200 (98.52)	174 (85.71)	97 (47.78)	32 (15.76)	118 (58.13)	21 (10.34)	58 (28.57)	2 (0.99)	162 (79.80)
^*a*^*P*-value		1.0000	1.0000	1.0000	1.0000	0.1627	0.0005	0.2904	0.5921	0.0013	0.0003	1.0000
MSSA	58	54 (93.10)	0	58 (100.00	19 (32.76)	11 (18.97)	12 (20.69)	33 (56.90)	0	11 (18.97)	0	43 (74.14)
Total	269	261 (97.03)	176 (65.43)	266 (98.89)	200 (74.35)	114 (42.38)	50 (18.59)	154 (57.25)	22 (8.18)	76 (28.25)	5 (1.86)	212 (78.81)
^*b*^*P-*value		0.0688	< 0.0001	1.0000	< 0.0001	< 0.0001	0.7033	1.0000	0.0057	0.0990	0.5883	0.3648

All isolates were susceptible to vancomycin and linezolid, and hence, not listed in the table

*PEN* Penicillin, *OXA* oxacillin, *ERY* Erythromycin, *CLI* Clindamycin, *TET* Tetracycline, *GEN* Gentamicin, *CHL* Chloramphenicol, *CIP* Ciprofloxacin, *RIF* Rifampin, *SXT* Trimethoprim-sulfamethoxazole

*MDR* multi-drug resistance, *MDR-MRSA* resistant to ≥3 classes of non-β-lactam antimicrobials, *MDR-MSSA* resistant to ≥3 classes of antibiotics including β-lactam antibiotics

^*a*^Comparison between *agr* dysfunctional and *agr* functional isolates among MRSA

^*b*^Comparison between MRSA and MSSA isolates

### *agr* sequencing

Two isolates that belonged to ST239 had no mutations in *agr* locus, and another ST239 isolate harbored a synonymous mutation in *agrA*. Inactivating mutations were detected in other 5 MRSA and 2 MSSA isolates. The variants were characterized by nonsynonymous changes (*n* = 5) and frameshift mutations (insertions, *n* = 2), which mainly occurred in *agrC* and *agrA* (Table 6).

**Table 6:** *agr* mutations for non-hemolytic isolates

Strain	MLST	*agr* mutations	Predicted results
R85^*a*^	59	*agrC* 62–63 ins t	Frameshift-truncated AgrC
R90^*a*^	59	*agrA* g443a	Asp>Gly at aa 148 in AgrA
11-3^*b*^	239	*agrD* a48g; *agrC* t884c	No aa change in *agrD*; Ile > Thr at aa 295 in AgrC
11-6^*b*^	239	None	None
11-15^*c*^	9	*agrB* g519a; Many mutations in *agrC* and *agrA*	No aa change in AgrB; Many aa changes in AgrC and AgrA
12-31^*b*^	239	None	None
12-98^*b*^	1296	*agrD* a7g; *agrC* a704c + a875g	Thr > Ala at aa 3 in AgrD; Tyr > Ser at aa 235 + Asn > Ser at aa 292 in AgrC
SA2017112^*b*^	239	*agrA* t615c	No aa change in AgrA
S14^*d*^	398	*agrC* c415t; *agrA* c263t	Pro>Ser at aa 139 in AgrC; Thr > Met at aa 88 inAgrA
S78^*e*^	22	*agrC* 434–435 ins t	Frameshift-truncated AgrC

^*a*^Compared to SA957 genome (NC_022442.1); ^*b*^Compared to NCTC8325 genome (CP000253.1); ^*c*^Compared to N315 genome (NC_002745.2); ^*d*^Compared to ST398 genome (NC_017333.1); ^*e*^Compared to H-EMRSA-15 genome (CP007659.1). ins, insertion; del, deletion; aa, amino acid; −, not applicable

## Discussion

Recent evidence indicated that *agr* dysfunction was common among *S. aureus* clinical isolates, especially MRSA [11–14, 17, 18]. However, the dysfunction was unusual in pediatric populations in China according to the current study. Moreover, the data showed that infections caused by *agr*-dysfunctional strains were always HA. Similarly, the study by Shopsin et al. [35] showed that the carriage of an *agr*-defective strain was associated with hospitalization. Butterfield et al. [13] also indicated that *agr* dysfunction was closely associated with prior administration of β-lactam and fluoroquinolone. Therefore, the high antibiotic selection pressure in the medical environment might lead to the emergence of *agr* dysfunction, which might be due to the *agr*-controlled virulence that is energy-consuming and needs to be balanced with the expression of antibiotic resistance in a healthcare environment filled with antibiotics [36].

In the current study, MRSA strains showed strong homology, and ST59-SCC*mec* IV and ST239-SCCmec III were the most prevalent clones. This result was consistent with that of a previous study conducted by Qiao et al. in Chinese children [37]. These results showed that *agr* dysfunction was more common in ST239-SCC*mec* III than in ST59-SCC*mec* IV isolates, the phenomenon was consistent with a previous study, which indicated that SCC*mec* IV/IVa MRSA (3%, known as CA-MRSA clone) was associated with lower rates of *agr* dysfunction as compared to SCC*mec* I-III MRSA (43%, known as HA-MRSA clone) [38]. Thus, the low prevalence of *agr* dysfunction in this study might be attributed to more than half of the MRSA isolates belonging to ST59 harboring the SCC*mec* IV.

In addition, infections caused by PVL-producing ST22-t309-*agr* I clone should be under intensive research. The current literature demonstrated that ST22-MRSA isolates mainly carry SCC*mec* IV [39, 40]. However, in this study, ST22-t309-*agr* I-MRSA strains were classified as SCC*mec* V. Moreover, ST22-t309-*agr* I present in MSSA isolates indicated that ST22-t309-MRSA probably arose from ST22-t309-MSSA.

Furthermore, *S. aureus* can lead to chronic infections by forming the biofilm on the surface of medical implants [41]. Herein, a majority of the *S. aureus* isolates were strong biofilm formers, especially MRSA, which should be brought to the attention of Chinese pediatricians. In addition, *agr* dysfunction has been linked to increased biofilm formation and enhanced colonization ability previously [11]; however, this phenomenon was inconsistent with that in the current study and could be attributed to the small sample size of *agr* dysfunctional isolates.

Both PVL and biofilm are major virulence factors of *S. aureus*. Previous studies found that some secreted virulence factors are closely related to biofilm formation, such as phenol-soluble modulin α (PSMα) [42]. Thus, we tried to explore the correlation between PVL and biofilm formation. However, no such association was found in both MRSA and MSSA isolates. A previous meta-analysis demonstrated that PVL strains are rare in colonizing isolates as compared to isolates causing skin and soft-tissue infections [43]. These results indicated that PVL was primarily associated with disease rather than biofilm formation and colonization.

Intriguingly, we found that *agr* dysfunctional isolates were more resistant than isolates with functional *agr.* However, at present, *agr* dysfunction is the result or cause of drug resistance remains unclear, while methicillin resistance might lead to *agr* dysfunction. Rudkin et al. [44] found that the expression of *mecA* (major resistance determinant of MRSA) could subtly affect the peptidoglycan structure or its interaction with other cell wall-associated proteins and prevented the detection of autoinducing peptide (AIP), thereby resulting in an unresponsive *agr* system and the subsequent low-level toxicity. When the type II SCC*mec* element or only *mecA* was deleted from an isolate with dysfunctional *agr*, the *agr* activity was restored. However, Tsuji et al. [45] suggested that *agr* dysfunctional might directly influence the acquisition of intermediate resistance to vancomycin after subtherapeutic exposure.

Nonetheless, all *S. aureus* strains, including the 10 *agr* dysfunctional isolates, were sensitive to vancomycin. Kim et al. [46] showed that 2/12 initial *agr*-functional isolates acquired *agr* dysfunction during vancomycin therapy for persistent bacteremia, but were still sensitive to vancomycin. In addition, all the 4 strains developed from vancomycin-susceptible *S. aureus* (VSSA) to heterogeneous VISA (hVISA) were initially *agr* dysfunctional strains. Therefore, we speculated that *agr* dysfunctional isolates can more easily adapt to glycopeptide selection pressure than *agr* functional isolates. Taken together, further studies assessing the correlation between *agr* dysfunction and antibiotic resistance, especially vancomycin resistance, are imperative.

The *agr* quorum sensing system has become a new target for developing new antibiotics. Hitherto, many natural and synthetic compounds have been found to interfere with the functions of *agr* [47]. However, the prevalence of *agr* dysfunction among *S. aureus*, and the potential correlation between *agr* dysfunction and antibiotic resistance indicated that isolates could withstand drug interference with *agr* functionality emerge rapidly. Strikingly, the promotion effects of *agr* dysfunction on biofilm formation may pose a great threat for patients using indwelling devices. Thus, additional studies are needed to explore the feasibility of *agr* system as a new target for antimicrobial agents.

Herein, we found that the *agr* sequence was not only associated with the *agr* group but also to the MLST types. Thus, the *agr* group and MLST types were considered while detecting the occurrence of mutation in the current *agr* dysfunctional isolates. Furthermore, *agr* dysfunctional isolates detected mutations that mainly occurred in *agrC* and *agrA*, leading to the inactivation of the auto-activation circuit and *RNAIII* expression [48]. However, no mutation or synonymous mutation or no mutations were also detected in our study, which suggested the involvement of additional mechanisms in *agr* dysfunction. Interestingly, *mecA* expression [49, 50] and abnormal expression of regulators of *agr* (*sarA*) can also be the cause of *agr* dysfunction [11].

Nevertheless, the current study had limitations. First, the sample size was relatively small. Second, this was a single-center study, and all the isolates were collected from one hospital. However, the hospital serves the whole of China, and > 60% of the hospitalized children come from all over the country. Therefore, the present study is still representative in China. Third, some MRSA strains used in this study have been sub-cultured 2 or 3 times since first collected, and multiple subcultures and long-term cryopreservation might have affected the characteristics of the strain.

## Conclusions

In summary, *agr* dysfunction was not common in pediatric populations in China, and ST59-SCC*mec* IV was associated with a low prevalence of *agr* dysfunction as compared to the ST239-SCC*mec* III isolates. *Agr* dysfunctional isolates were always healthcare-associated and multidrug resistant. Except for the gene mutations, other mechanisms might also be involved in *agr* dysfunction. The *agr* dysfunction might be a major adaption mechanism of *S. aureus* to antibiotic selection pressure. Thus, an in-depth understanding of *agr* dysfunction is an urgent requirement for the development of new antibiotics that target *agr* expression.

## Abbreviations

*agr*: accessory gene regulator
AI: Intra-abdominal infection
BJI: Bone and joint infection
BSI: Bloodstream infection
CA: Community-associated
CHL: Chloramphenicol
CIP: Ciprofloxacin
CLI: Clindamycin
CLSI: Clinical and Laboratory Standards Institute
CNSI: Central nervous system infection
ERY: Erythromycin
GEN: Gentamicin
HA: Healthcare-associated
hVISA: heterogeneous VISA
IE: Infective endocarditis
LA-MRSA: Livestock-associated MRSA
LNZ: Linezolid
MDR: Multidrug resistance
MLST: Multilocus sequence typing
MRSA: Methicillin-resistant *S. aureus*
MSCRAMMs: Microbial surface components recognizing adhesive matrix molecules
OD: Optical density
ODc: Cut-off OD value
OXA: Oxacillin
PCR: Polymerase chain reaction
PEN: Penicillin G
*pvl*: panton-Valentine leukocidin
RIF: Rifampin
*S. aureus*: *Staphylococcus aureus*
SCC*mec*: Staphylococcal cassette chromosome *mec*
SP: Severe pneumonia
*spa*: staphylococcal protein A
SSTI: Skin and soft tissue infection
SXT: Sulphamethoxazole/trimethoprim
TCP: Tissue culture plate method
TET: Tetracycline
TSB: Tryptic soy broth
VAN: Vancomycin
VISA: Vancomycin intermediate *S. aureus*
VSSA: Vancomycin-susceptible *S. aureus*
